# Supplementary figures and images for: Effectiveness and Factors Associated with Improved Life Skill Levels of Participants of a Large-Scale Youth-Focused Life Skills Training and Counselling Services Program (LSTCP): Evidence from India
Source: Behav Sci (Basel). 2022 Jun 15;12(6):191. doi: 10.3390/bs12060191 (PMC9220777; doi:10.3390/bs12060191)

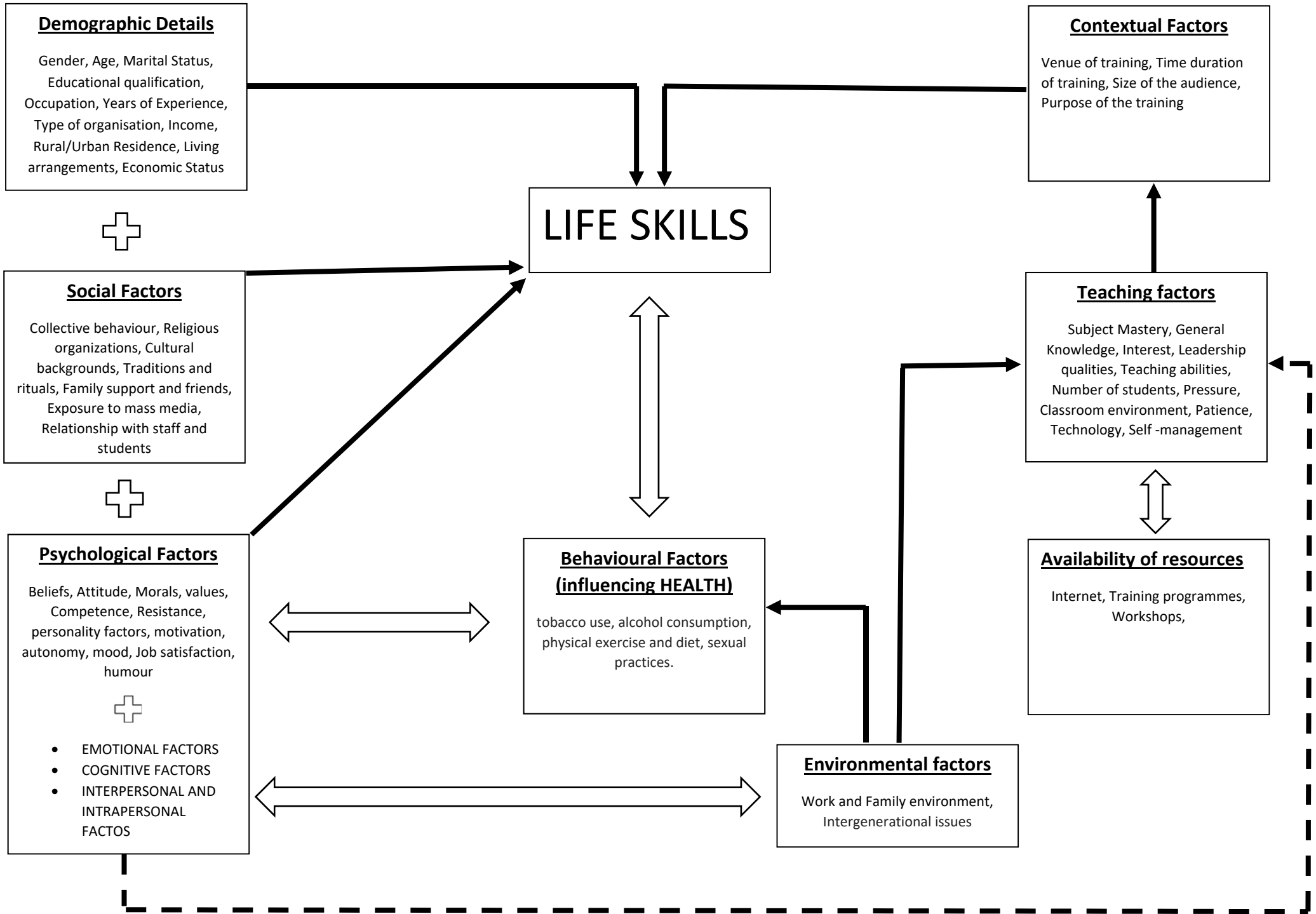

Supplement: Supplementary file 1 [file behavsci-12-00191-s001.zip › behavsci-1721439-supplementary.pdf]
